# Exploring the elevation dynamics of rumen bacterial communities in Barn feeding cattle from 900 to 3,600 meters by full-length 16S sequencing

**DOI:** 10.3389/fvets.2023.1169573

**Published:** 2023-07-17

**Authors:** Shuli Yang, Jieyi Zheng, Shichun He, Zaimei Yuan, Rongjiao Wang, Dongwang Wu

**Affiliations:** ^1^Guangdong Provincial Key Laboratory of Animal Molecular Design and Precise Breeding, College of Life Science and Engineering, Foshan University, Foshan, China; ^2^Key Laboratory of Animal Nutrition and Feed Science of Yunnan Province, Yunnan Agricultural University, Kunming, China; ^3^Kunming Animal Disease Prevention and Control Center, Kunming, China; ^4^Panzhihua Academy of Agricultural and Forestry Sciences, Panzhihua, China

**Keywords:** rumen microbiology, cattle, microbiome, bacteria, altitude

## Abstract

The diversity and abundance of rumen microorganisms serve as indicators not only of the host’s digestive and metabolic capacity but also of its health status. The complex microbial communities in the rumen are influenced to varying degrees by environmental adaptability. In this study, we collected 24 rumen fluid samples from 24 healthy male cattle in three regions of Yunnan, China. Using 16S rRNA amplicon sequencing data analysis, we examined the variations in rumen microorganisms among cattle fed at altitudes of 900 m, 1800 m, and 3,600 m. Altitude-related environmental factors did not surpass phylogeny as the main driving force behind the convergent evolution of yellow cattle rumen microbiome composition. However, they did have an impact on the alpha diversity of the rumen microbiome and the coevolution of the core microbiome. The change in altitude noticeably influenced the diversity and richness of the rumen microbiota, highlighting the environmental effect of altitude. As altitude increased, there was an observed increase in the abundance of *Firmicutes* and *Bacteroidetes*, while the abundance of ruminal *Proteobacteria* and *Kiritimatiellaeota* decreased. Importantly, at the genus level, the core genus exhibited distinct dynamic changes as altitude increased. Ruminants exhibit the ability to adapt their gut type in accordance with altitude, thereby optimizing energy utilization, especially in high-altitude settings. These discoveries offer valuable insights into the coevolution of host–microbe interactions during ruminant adaptation to various altitudinal environments.

## Introduction

The gut of animals harbors a vast population of microbes, and a growing body of research indicates that the intestinal flora is extensive and vital for animal nutrition and health (1–4). Alterations in the composition of gut microbiota can influence host phenotypes associated with digestion, development, immunity, and behavior (5). Bioactive metabolites produced by the intestinal flora influence host physiological processes, immune system regulation, and hormone secretion (6, 7). The composition, diversity, and function of the microbial community are closely associated with factors such as animal species, diet, environment, and other variables (8–10). The complex interaction of the host genome, nutrition, and living environment governs the composition and activity of the intestinal flora (11). The interactions shape the functional composition of intestinal flora species and contribute to the response to environmental stress. The impact of animal intestinal microecology on host physiology has long been a focal point of ecological research, particularly under changing environmental conditions. For example, despite the challenging conditions encountered at high altitudes, many animals thrive and develop specific physiological mechanisms. The intestinal flora may play a crucial role in adapting to the plateau environment (12). Certain studies suggest that gut microbes play a role in helping animals adapt to high altitudes. The rumen microbial genes of yaks and sheep at high altitude showed a significant enrichment in the volatile fatty acid production pathway, while the rumen microbial genes of cattle at low altitude displayed an enrichment in the methanogenesis pathway (13). Pikas that have adapted to the cold and low-oxygen high-altitude environment at high altitudes demonstrated higher intestinal microbial diversity, volatile fatty acid concentration, and cellulose degradation ability compared to the pikas residing in low-altitude areas (14). Research findings indicate that rhesus monkeys in high-altitude environments exhibit a higher abundance of Firmicutes to Bacteroidetes in their intestinal flora, along with an elevated presence of ruminococcaceae and Christensenellaceae. These factors potentially contribute to their adaptation to high altitudes (15).

Yellow cattle exhibit remarkable adaptability to various altitude environments, making them an ideal model for exploring the co-adaptation between extreme plateau environments and altitude gradients. As a result, they offer an opportunity to investigate the impact of varying altitudes on the composition and functionality of intestinal flora abundance. Currently, there is limited research on the interaction between rumen microflora and hosts in ruminants at different altitudes. In our previous study, we observed significant effects of altitude on the rumen microbes of yaks (16). The objective of this study is to investigate significant variations in the rumen microbiota of cattle residing at different altitudes, thereby enhancing our understanding of how the rumen microbiota influences host adaptation to distinct habitats. The findings will provide valuable reference information for research in microbial medicine conducted in high-altitude environments.

## Materials

All animals involved in this experiment have received approval from the Animal Protection and Utilization Committee of Yunnan Agricultural University, China, and have adhered to the guidelines of the Laboratory Animal Ethics Committee. The collection of experimental animal sources and samples was conducted in accordance with these regulations. Group H (*n* = 6) was located in the pasture of Tiancheng Lun Zhu Agricultural Products Development Co., Ltd., in the north of Shangri-La County. The experimental site had an average altitude of 3,600 meters and belonged to a temperate monsoon climate. The maximum average daily temperature was 13°C, the minimum average daily temperature was 1°C, the annual precipitation was 600 mm, and the relative humidity was 65%. Group L (n = 6) was situated in Jiangcheng Xinfutai Agricultural Development Co., Ltd., located in the west of Jiangcheng County. The average altitude of the site was 900 meters, and it belonged to a subtropical mountain monsoon humid climate. The average annual temperature was 18.1°C. Group M (n = 12) was positioned in Jinjiang Green Beef Cattle Breeding Co., LTD, in the southern part of Anning City. The site had an altitude of 1800 meters and experienced a subtropical climate. The annual average temperature was 14.9°C, with extreme maximum and minimum temperatures of 31.5°C and − 7.8°C, respectively. All three experimental groups were fed in barns with a diet consisting of whole silage maize and Milling Corn, as outlined in Table 1, which provides information about the dietary composition and nutritional levels. Table 2 presents the effects of different elevations on yellow cattle fattening. The feeding period lasted for 90 days, during which the animals’ weights were measured on the first and last days before morning feeding. Two hours after the final morning feed, a catheter was inserted into the rumen, and rumen fluid samples were collected using a vacuum sampler. For each animal, 30 mL of rumen fluid was collected and divided into three parts, each placed in a 10 mL polypropylene tube and rapidly stored in liquid nitrogen. The samples were transported to the laboratory and stored in a refrigerator at −80°C.

**Table 1:** Nutrient composition of whole corn silage (dry matter basis except for dry matter conten that is fresh basis).

Diet	Items	Nutrient ratio
Whole plant corn silage	Dry matter (%)	45.73
	Ash content (%)	7.40
	Crude protein (%)	15.83
	Crude fat (%)	3.16
	Acid Detergent Fiber (%)	33.21
	Neutral Detergent Fiber (%)	59.03
	crude fibre (%)	10.20
	Calcium (%)	1.14
	phosphorus (%)	0.27

**Table 2:** Effects of different elevations on yellow cattle fattening.

Ration level	Low altitude group (L)	Medium altitude group (M)	High altitude group (H)
DMI (kg/d)	6.80 ± 0.01	6.74 ± 0.14	5.75 ± 0.09
Initial Weight(kg)	229.08 ± 38.62	237.08 ± 44.53	163.71 ± 17.62
Fattening Period(d)	90	90	90
Final Weight(kg)	312.33 ± 44.99	318.08 ± 48.08	236.92 ± 15.04
Total weight gain during fattening(kg)	83.25 ± 13.45	81.00 ± 10.55	73.21 ± 12.68
ADG (kg/d)	0.93 ± 0.15	0.90 ± 0.12	0.81 ± 0.14

### DNA extraction and sequencing

The microbial community DNA was extracted using the EZNA Stool DNA Kit (Omega Bio-Tek, Norcross, Georgia, United States), following the manufacturer’s instructions. The DNA was quantified using a Qubit Fluorometer and the Qubit dsDNA BR Assay kit (Invitrogen, USA), and the quality was assessed by running an aliquot on a 1% agarose gel. The variable regions V1–V9 of the bacterial 16S rRNA gene were amplified using degenerate PCR primers, 27F (5’-AGRGTTYGATYMTGGCTCAG-3′) and 1492R (5’-RGYTACCT TGTTACGACTT-3′) (17). Both the forward and reverse primers were tagged with Illumina adapters, pad, and linker sequences. PCR enrichment was carried out in a 50 μL reaction containing 30 ng of template, fusion PCR primer, and PCR master mix. The PCR cycling conditions were as follows: 94°C for 3 min, followed by 30 cycles of 94°C for 30 s, 56°C for 45 s, and 72°C for 45 s, with a final extension at 72°C for 10 min. The PCR products were purified using AmpureXP beads and eluted in Elution buffer. The libraries were assessed using the Agilent 2,100 bioanalyzer (Agilent, United States). The validated libraries were sequenced on the Illumina MiSeq platform (BGI, Shenzhen, China) using the standard Illumina pipelines, generating 2 × 300 bp paired-end reads.

### Sequence analyses

The raw data were filtered to eliminate adapter contamination and low-quality readings, resulting in clean reads. The paired-end reads with overlaps were then merged to form tags. These tags were subsequently clustered into Operational Taxonomic Units (OTUs) at a 97% sequence similarity. Taxonomic ranks were assigned to representative sequences of the OTUs using the Ribosomal Database Project (RDP) Naive Bayesian Classifier v.2.2. Alpha diversity, beta diversity, and the identification of different species were analyzed based on the OTUs and taxonomic ranks. The clustering of tags into OTUs was performed using USEARCH (v7.0.1090) software. The taxonomic classification of the OTU representative sequences was done using the Ribosomal Database Project (RDP) Classifier v.2.2 trained on the Greengene_2013_5_99 database, with a cutoff confidence value of 0.5. The filtered tags were clustered into OTUs at 97% similarity. The number of OTUs per sample primarily represents the sample’s diversity level. The OTUs of each group were listed, and Venn diagrams were created using the Venn Diagram software in R (v3.1.1) to summarize the common and specific OTU IDs.

Based on the abundance information of the OTUs, the relative abundance of each OTU in each sample was calculated. Principal Component Analysis (PCA) of the OTUs was performed using the relative abundance values with the ade4 package in R (v3.1.1). Good’s coverage, alpha diversities (including Inverse Simpson and Shannon indices), richness (observed number of OTUs), and evenness (Shannon evenness) were calculated using Mothur V.1.31.2. Beta diversity analysis was conducted using QIIME (v1.80). Since there were differences in sequencing depth among the samples, normalization was introduced by randomly extracting sequences according to the minimum sequence number across all samples. The extracted sequences formed a new ‘OTU table biom’ file, and the beta diversity distance was calculated based on this file. Statistical results, including beta diversity differences between groups, species abundance histograms, and histograms comparing differences in key species, were plotted using R (v3.4.1). Bacterial community typing was conducted using R (v3.4.1). KEGG function prediction was performed using R (v3.2.1) and the software PICRUSt2 v2.3.0-b. The LEfSe software was utilized for differential species analysis.

## Results

### Analysis of rumen microbial diversity

**Figure 1:**
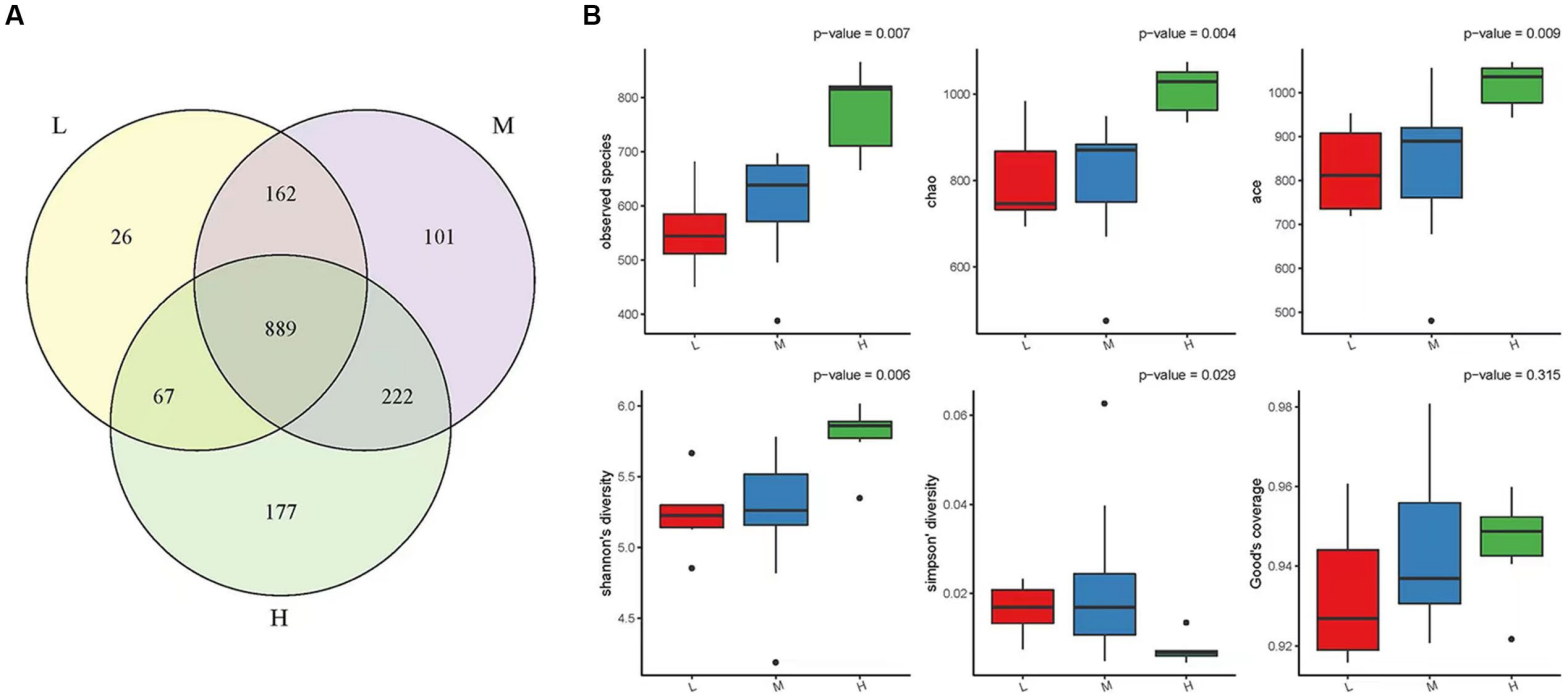
**(A)** OTU Venn diagram. In the Figureure, different color graphs represent different samples or different groups, and the number of overlapping parts is the number of OTUs shared between two samples or two groups. Similarly, the number of multiple overlapping parts refers to the number of OTUs shared among multiple samples or groups. Low altitude group (L), medium altitude group (M), high altitude group (H). **(B)** Alpha diversity box chart. The Observed Species index, Chao index, ACE index, Shannon index, Simpson index and Good-coverage index are included. The larger the first four indices, the smaller the fifth index, the more abundant the species in the sample. Low altitude group (L), medium altitude group (M), high altitude group (H).

A total of 1,644 OTUs were identified in the three experimental groups: high altitude, middle altitude, and low altitude. The high altitude group had 1,355 OTUs, the middle altitude group had 1,374 OTUs, and the low altitude group had 1,144 OTUs. As shown in Figure 1A, a total of 889 OTUs were present in the three experimental groups, with 177 OTUs unique to the high-altitude group, 101 OTUs unique to the medium-altitude group, and 26 OTUs unique to the low-altitude group.

Alpha diversity was evaluated using parameters such as the Observed species index, Chao index, ACE index, Shannon index, Simpson index, and Good-coverage index based on abundance (Figure 1B). The Observed species index, Chao index, ACE index, and Shannon index showed an increasing trend in the low, middle, and high altitude groups, indicating that the diversity and richness of rumen microbiota in the high altitude group were the highest (*p* < 0.05). Moreover, the Simpson index, which reflects the species diversity of the communities, showed that the diversity of rumen microorganisms in the high-altitude group was higher than that in the medium-low altitude group.

**Figure 2:**
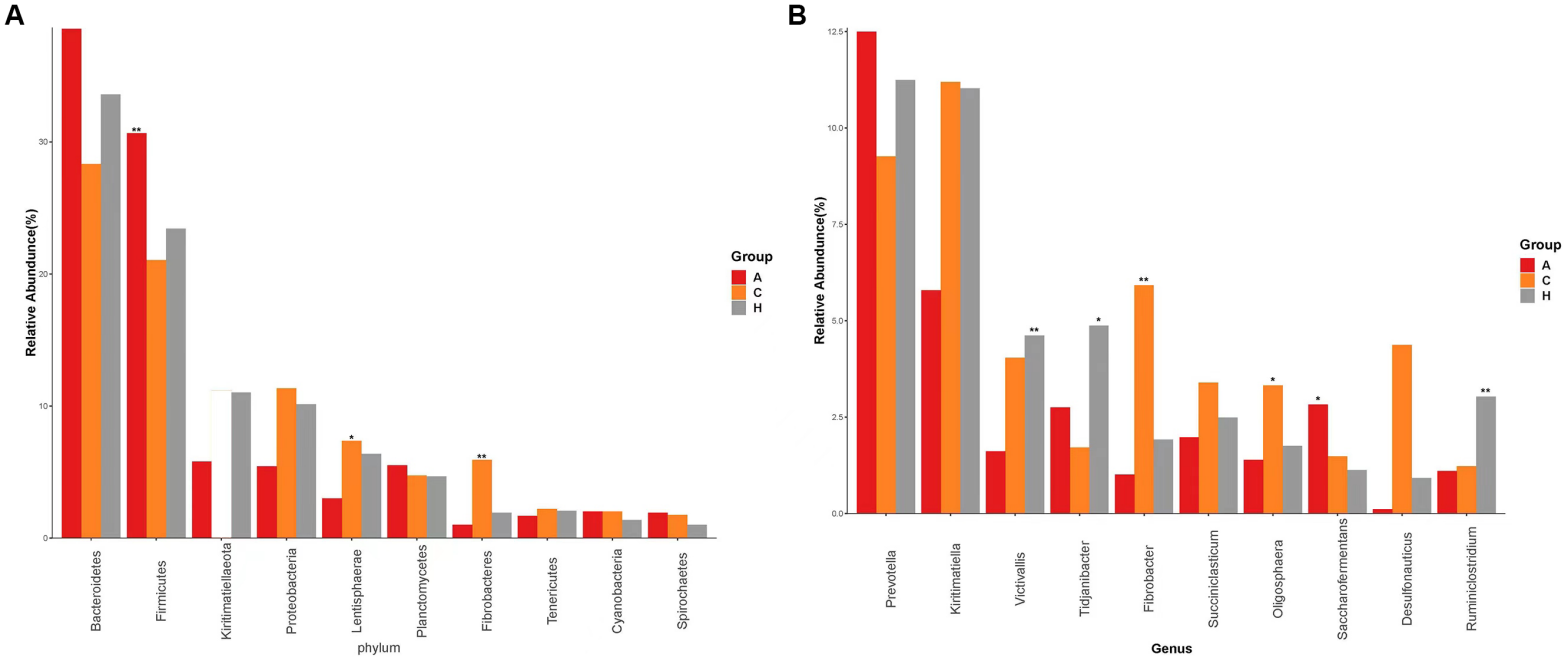
Species with the top 10 abundence, showing the average relative abundence of each group and the significance of the difference test (marked with an “*” at the top of the bar chart if any, not marked if not). **(A)** Comparison of dominant phyla in Low altitude group, Medium altitude group, High altitude group. **(B)** Comparison of dominant genera in the Low altitude group, Medium altitude group, High altitude group. Low altitude group (L), medium altitude group (M), high altitude group (H).

We detected 19 phyla (Supplementary Figure S1A) in the samples from the three elevation regions, which accounted for more than 0.1% of the community abundance at the phylum level. The dominant phyla were Bacteroidetes, Firmicutes, Proteobacteria, and Kiritimatiellaeota. The relative abundance of Firmicutes and Bacteroidetes was 21.55, 23.41, and 31.03% at low, middle, and high altitudes, respectively, while Kiritimatiellaeota had relative abundances of 28.62, 34.64, and 38.52% at the same altitudes. This trend indicated an obvious increase in relative abundance with increasing altitude (Figure 2). The ratio of Firmicutes to Bacteroidetes was 0.75, 0.67, and 0.80 in yellow cattle at low, middle, and high altitudes, respectively.

The abundance of Proteobacteria and Kiritimatiellaeota in the rumen exhibited a decrease as altitude increased. Additionally, the relative abundance of Firmicutes, Lentisphaerae, and Fibrobacteres showed significant differences among yellow cattle rumen microorganisms at low, medium, and high altitudes. In the 24 samples analyzed (Supplementary Figure S1B), we identified 33 genera, with *Prevotella* and *Kiritimatiella* being the most abundant across all three elevation levels in the rumen of yellow cattle. Notably, there were distinct and dynamic changes observed at the genus level in ruminal bacteria as altitude increased.

### Elevation environment and differential microbes

**Figure 3:**
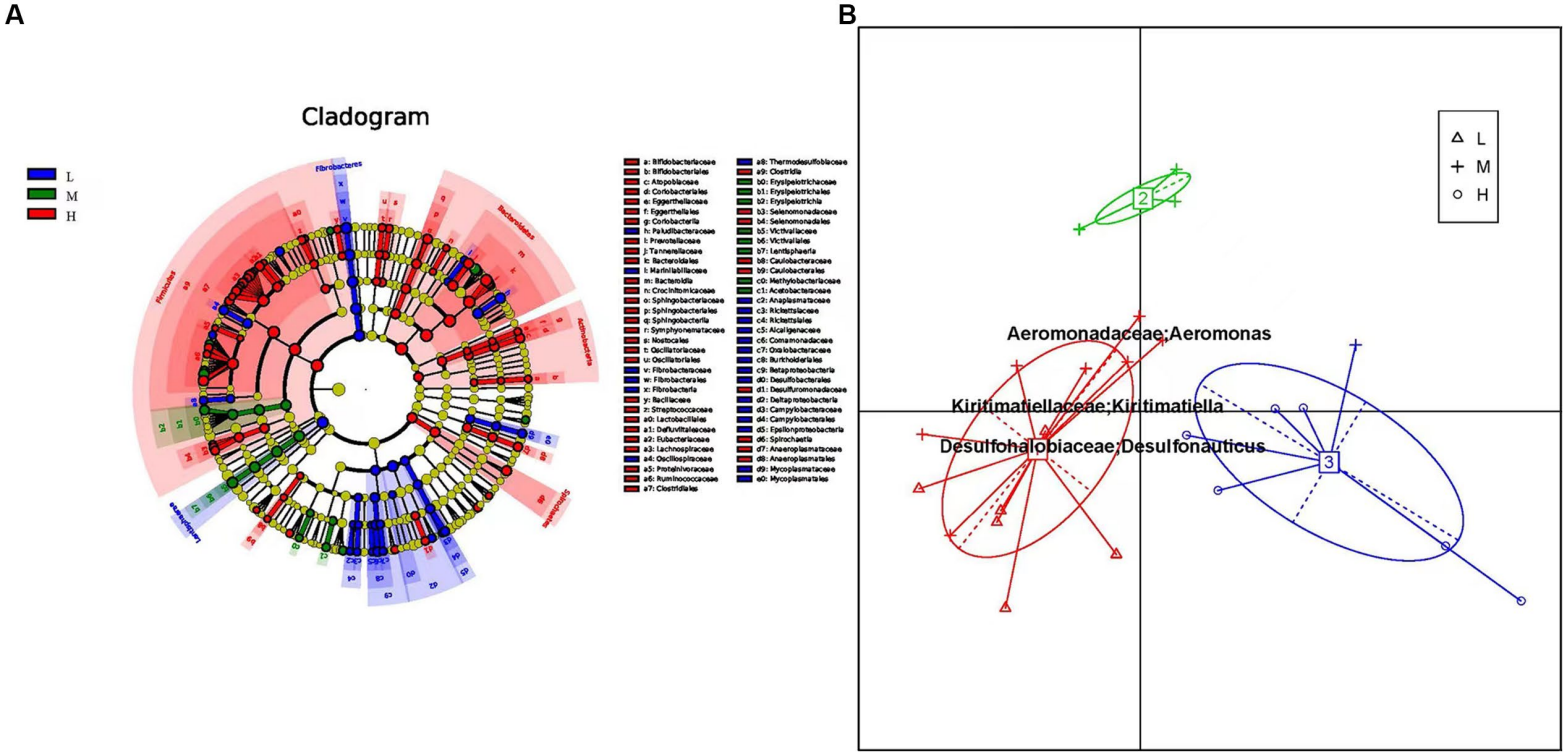
**(A)** Cluster plots were analyzed by LEfSe. Different colors represent different groups, nodes of different colors represent the microbiota that play an important role in the group represented by the color, a color circle represents a biomarker, and the legend in the upper right corner is the biomarker name. Yellow nodes indicate microbial taxa that did not play an important role in the different groupings. From the inside to the outside, each circle is divided into phylum, class, order, family, and genus level species. **(B)** Enterotypes analysis. The abscissa represents principal component one, and the ordinate represents principal component two, which are the two principal components with the largest variance contribution rate.

The prokaryotic community composition in the rumen exhibits significant variations at both the phylum and genus levels. To investigate the differential microbial communities among the low, middle, and high altitude groups, we utilized linear discriminant effect sizes (LEfSe) analysis, including LDA (linear discriminant analysis) (Figure 3A). The LEfSe results showed that microbial groups with significant effects were displayed in different colors in the low, middle, and high altitude groups. Among these groups, the high altitude group had the largest number of different microorganisms (Supplementary Figure S2). The signature gut microbiota in the low-altitude group included Fibrobacteria, Fibrobacteraceae, and Lentisphaerae. Victivallaceae was predominant in the medium-altitude group, while Bacteroidetes and Clostridial were prominent in the high-altitude group. Considering the reports suggesting that intestinal type can reflect functional differences, we investigated whether the rumen bacterial community of yellow cattle could be categorized into functional groups based on altitude variations. Principal component analysis (PCA) revealed distinct intestinal types formed by the samples through Bray-Curtis differential analysis. Each cluster was characterized by changes in the abundance of its representative genus, Enterotype 1 exhibited Kiritimatiella and Desulfonauticus, while Enterotype 2 showed a high abundance of Aeromonas (Figure 3B).

### Predicted function and metabolism of rumen microbiota

**Figure 4:**
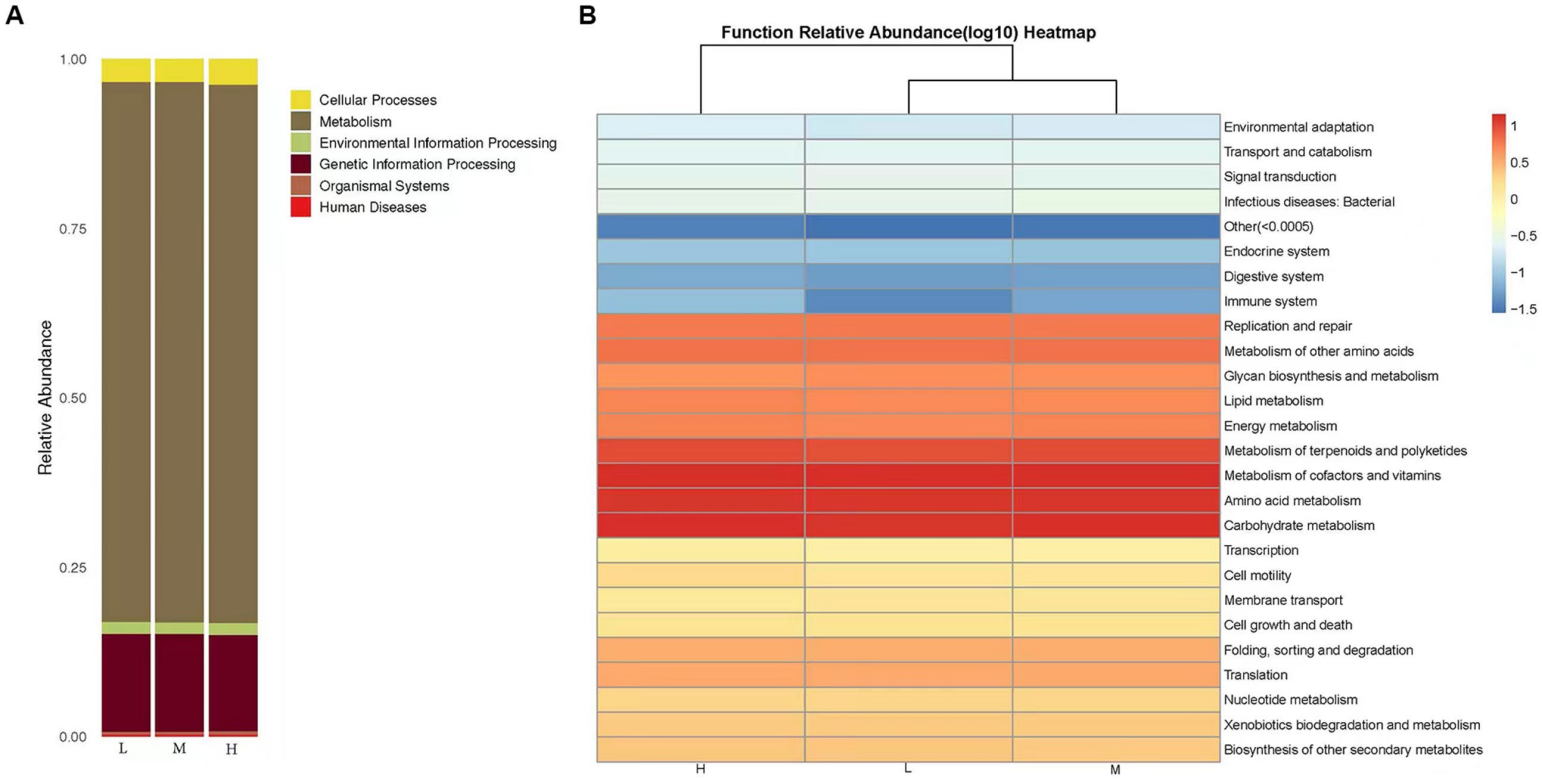
**(A)** KEGG classification bar chart. The predicted microbiota function based on the KEGG database, the horizontal axis represents the sample, and the vertical axis represents the relative abundance of the predicted function. **(B)** Heat map of functional predicted abundance. Longitudinal clustering indicates the similarity of functional prediction among different samples. The closer the distance, the shorter the branch length, indicating that the functional prediction and abundance of samples are more similar. Horizontal clustering indicates that the abundance of this function is similar in each sample.

The predictions of bacterial community KEGG function abundance were obtained using PICRUST2. In the low, middle, and High altitude groups, the relative abundances of Metabolism and Genetic Information Processing were 79.72 and 14.44%, respectively (Figure 4A). A total of 29 biochemical pathways were identified among the metabolic functions. Functions related to Metabolism of cofactors and vitamins, Carbohydrate metabolism, and Amino acid metabolism were enriched in all samples (Figure 4B). Additionally, the microflora of the low, middle, and high altitude groups exhibited other functional roles, such as cellular processes, organismal systems, environmental information processing, human diseases, and genetic information processing.

## Discussion

Animal gut microbes are influenced by various factors, including diet, genetics, age, environment conditions such as altitude and geographical location (18–21). This study focuses on exploring the relationship between rumen bacterial composition and function in Yellow cattle with respect to altitude. Gut microbes play an important role in host adaptation to different diverse environments (22, 23), providing essential nutrients and maintaining intestinal homeostasis (24, 25). Previous studies have demonstrated that different elevations have specific effects on the composition and fermentation function of rumen microbiomes in grazing yaks (26). Furthermore, it has been observed that the altitude environment drives convergent evolution of α diversity and indicator microbiota in animal gut microbiota (27). The intestinal microbiota of hosts exhibits distinct characteristics according to different altitude habitats (28). The shared features of intestinal microbiota at various elevations suggest a co-evolution between mammalian gut microbiota and their hosts (29). Numerous studies have demonstrated that alterations in altitude can influence the changes in intestinal microecology, subsequently impacting the structure and function of mammalian intestinal flora (30). Intestinal population diversity is profoundly influenced by altitude, with notable distinctions observed between high altitude and low altitude populations. The intestinal microbial communities in yaks at different altitudes were dominated by Firmicutes (63.42%) and Bacteroidetes (47.4%) at the phylum level (16). Due to cold stress, ruminants at high altitudes may experience an increased reliance on carbohydrates, necessitating Firmicutes and Bacteroidetes to supply additional energy for maintenance purposes.

The interaction between intestinal flora and host not only regulates metabolism, but also serves as a crucial bridge connecting the environment and host, thus helping the host better adapt to different environments (17). The diversity analysis revealed an increasing trend in both the diversity and uniformity of rumen bacteria among cattle in low, middle and high altitude areas. In this study, Yellow cattle from all altitude regions were fed the same diet to maintain uniformity, highlighting altitude as the primary factor influencing the changes in rumen microbial diversity. Previous studies have demonstrated higher rumen bacterial community diversity and rumen fluid volatile fatty acid content (VFA) in yaks at an altitude of 4,700 m above sea level on the Qinghai-Tibet Plateau compared to those at middle and low altitudes (31). Through sample clustering, it was observed that the rumen bacteria of Yellow cattle at low, middle, and high altitudes did not primarily group together in the evolutionary branch, but rather individuals within the same altitude exhibited clustering. In terms of the number of endemic microorganisms, the number of endemic rumen bacteria at high altitude was significantly higher than that at middle and low altitudes, but most of the core microorganisms at the three altitudes were common. The co-evolution of the host-gut bacterial system has formed a common core microbe under the influence of different elevations (32).

The meadow at different elevations exhibit variations species richness and the forage found within them possesses varying nutritional value (33). As a result, the high-altitude group displayed significantly greater bacterial diversity compared to the low-altitude group. It is generally observed that higher gut bacterial diversity and richness are associated with a healthy and stable host gut microbiome (34). In contrast to the low altitude group, the high altitude group exhibited a higher abundance of Bacteroidetes and Firmicutes in the rumen. These bacteria play a crucial role in the decomposition of fibers and cellulose, providing the necessary energy for the host (35). Furthermore, the high altitude group displayed a noticeable upward trend in the Firmicutes/Bacteroidetes ratio compared to the low and middle altitude groups. The elevated ratio of Firmicutes to Bacteroidetes in the rumen of the high altitude group indicates a greater propensity for fat deposition (36, 37). Studies have revealed a significant difference in the ratio of Firmicutes to Bacteroidetes in the gastrointestinal microbiome between high-altitude and low-altitude ruminants. The higher ratio observed in high-altitude ruminants has been shown to impact energy deposition (38). Altitude affects the energy metabolism of the gut microbiome and the ability to decompose substances such as fiber and cellulose (39). Kiritimatiellaeota is involved in the biosynthetic pathway of arginine and fatty acids, thereby utilizing nitrogen in food and producing energy (23). In this study, the abundance of rumen Kiritimatiellaeota in the middle-high altitude group was found to be significantly lower than that in the middle-low altitude group. Kiritimatiellaeota plays a particularly crucial role in the rumen of herbivores (40).

Compared to the high altitude group, the middle and low altitude groups exhibited greater activity in the biosynthetic pathways of arginine and fatty acid. In response to altitude fluctuations, the gut microbiome can adapt its metabolic rate and enhance the extraction of energy from complex carbohydrates, thereby promoting co-evolution between the host and the microbes. *Prevotella*, the genus with the highest abundance in the rumen across all altitude groups, signifies optimal digestive dynamics and contributes to intestinal homeostasis. Hence, a higher diversity of *Prevotella* and other fiber-degrading microorganisms enhances the microbiota’s ability to ferment, promoting gut health (41). Research has indicated that a high *Prevotella*-*Bacteroides* ratio can impact fiber digestion and glucose metabolism (42). The ratio of *Prevotella*-*Bacteroides* in the rumen of the middle-high altitude group was significantly higher than that of the low-altitude group. This long-established host-*Prevotella* symbiosis, developed through hundreds of thousands of years of coevolution, can result in compromised host-microbial interactions, consequently impacting host health.

The relative abundances of *Tannerella*, *Prevotella* and *Eubacterium* increased with increasing altitude. The host’s physiological responses to altitude, such as changes in immune function and metabolism, can impact the microbial community composition. In addition, Firmicutes/Bacteroidetes in the high altitude group showed an obvious upward trend compared with the low and middle altitude group. The ratio of Firmicutes to Bacteroidetes in the rumen of the high altitude group was higher, indicating better fat depositiony create an environment where *Tannerella*, *Prevotella*, and *Eubacterium* thrive and establish higher relative abundances compared to other microbial groups. The increase in the relative abundances of *Tannerella*, *Prevotella*, and *Eubacterium* with higher altitude can be attributed to a combination of environmental factors, host physiological adaptations. The decrease in the relative abundance of *Fibrobacter* and *Kiritimatiella* with increasing altitude could be attributed to changes in environmental conditions. On the other hand, the significantly higher relative abundance of *Butyrivibrio* in the high altitude group suggests its ability to adapt and thrive in the unique conditions found at higher altitudes, potentially influenced by both environmental factors.

This study provides valuable insights into intestinal flora and its functionality. We elucidate the rumen bacteria composition and functional genome information in farmed cattle across different altitudes. Among these findings, Kiritimatiellaceae intestinal types are predominantly observed in the low-to-mid-altitude group, while Desulfonauticus intestinal types are more concentrated in the high-altitude group, likely due to their adaptation to cold environments. Previous research has demonstrated that the proportion of dietary carbohydrate content in baboons directly influences the transformation of the host intestinal pattern. Therefore, changes in altitude-specific dietary protein and carbohydrate content may offer an intriguing explanation for the dynamics of enterotypes and assist in identifying the enterotype of high-altitude ruminants (43). In this study, the most notable evidence of elevation-induced changes in intestinal types was observed in type 2 and type 3 at middle and high altitudes, while type 1 remained stable at low altitudes. This finding suggests that the long-term co-evolution between the host and the environment contributes to distinct dynamics of intestinal types, playing a vital role in ruminant formation and adaptation to high-altitude extreme environments. The functional attributes of the intestinal microbiome govern the interactions between the host and the microbiome, ultimately shaping their mutual relationship (44). The findings from PICRUSt2 analysis revealed distinct variations in the rumen microflora of Yellow cattle across the three altitude regions, with metabolism being the most prominent and active function. Specifically, carbohydrate metabolism and amino acid metabolism were predominant. Interestingly, our study also uncovered a striking similarity in the functional genetic composition of rumen microbes among cattle at the three elevations. These results imply that rumen bacteria in cattle exhibit a heightened sensitivity to environmental adaptability compared to gut bacteria.

## Conclusion

Altitude environmental factors do not supersede phylogeny in driving the convergent evolution of the yellow cattle rumen microbiome composition. However, they do exert an influence on rumen microbiome alpha diversity and the coevolution of the core microbiome. Notably, certain key genera, including *Tannerella*, *Ruminobacter*, and *Prevotella*, demonstrate associations with the altitude environment. Our findings suggest that high-altitude regions provide a more favorable environment for rumen bacterial fermentation compared to low-altitude or medium-altitude areas. Furthermore, there may exist convergent evolution between the core microbiome and the host. These results indicate that rumen microorganisms in Yellow cattle from high-altitude areas have adapted to extreme environments, enabling them to maximize feed utilization efficiency.

## Data availability statement

The datasets presented in this study can be found in online repositories. The names of the repository/repositories and accession number(s) can be found in the article/Supplementary material.

## Ethics statement

All animals used in this experiment were approved by the animal protection and utilization committee of Yunnan Agricultural University, China (protocol # 2018–009), and there was compliance with the guidelines of the Laboratory Animal Ethics Committee in experimental animal handling.

## Author contributions

DW and SY made substantial contributions to the conception or design of the experiments. SY and ZY performed the experiments. RW and SH analyzed the data. DW and ZY wrote the paper. All authors contributed to the article and approved the submitted version.

## Funding

This research was supported by the Science Research Foundation of Education Department of Yunnan Province (2023 J0518), National Natural Science Foundation of China (32060762), Doctoral Research Foundation of Yunnan Agricultural University (KY2022-53), Research Project of Department of Education of Guangdong Province (2022ZDZX4041), and Yunnan Agricultural Fundamental Research Projects (202301BD070001-095).

## Conflict of interest

The authors declare that the research was conducted in the absence of any commercial or financial relationships that could be construed as a potential conflict of interest.

## Publisher’s note

All claims expressed in this article are solely those of the authors and do not necessarily represent those of their affiliated organizations, or those of the publisher, the editors and the reviewers. Any product that may be evaluated in this article, or claim that may be made by its manufacturer, is not guaranteed or endorsed by the publisher.

## Supplementary material

The Supplementary material for this article can be found online at: https://www.frontiersin.org/articles/10.3389/fvets.2023.1169573/full#supplementary-material
