# Supplementary material for: Exploring the elevation dynamics of rumen bacterial communities in Barn feeding cattle from 900 to 3,600 meters by full-length 16S sequencing
Source: Front Vet Sci. 2023 Jul 17;10:1169573. doi: 10.3389/fvets.2023.1169573 (PMC10390322; doi:10.3389/fvets.2023.1169573)
Supplement: Supplementary file 1 [file Data_Sheet_1.docx]

Supplementary Material

Exploring the elevation dynamics of rumen bacterial communities in Barn feeding cattle from 900 to 3600 meters by full-length 16S sequencing

**Shuli Yang, Jieyi Zheng, Shichun He, Zaimei Yuan, Rongjiao Wang, Dongwang Wu***

*** Correspondence:** Dongwang Wu, danwey@163.com

# Supplementary Figures and Tables

## Supplementary Figures


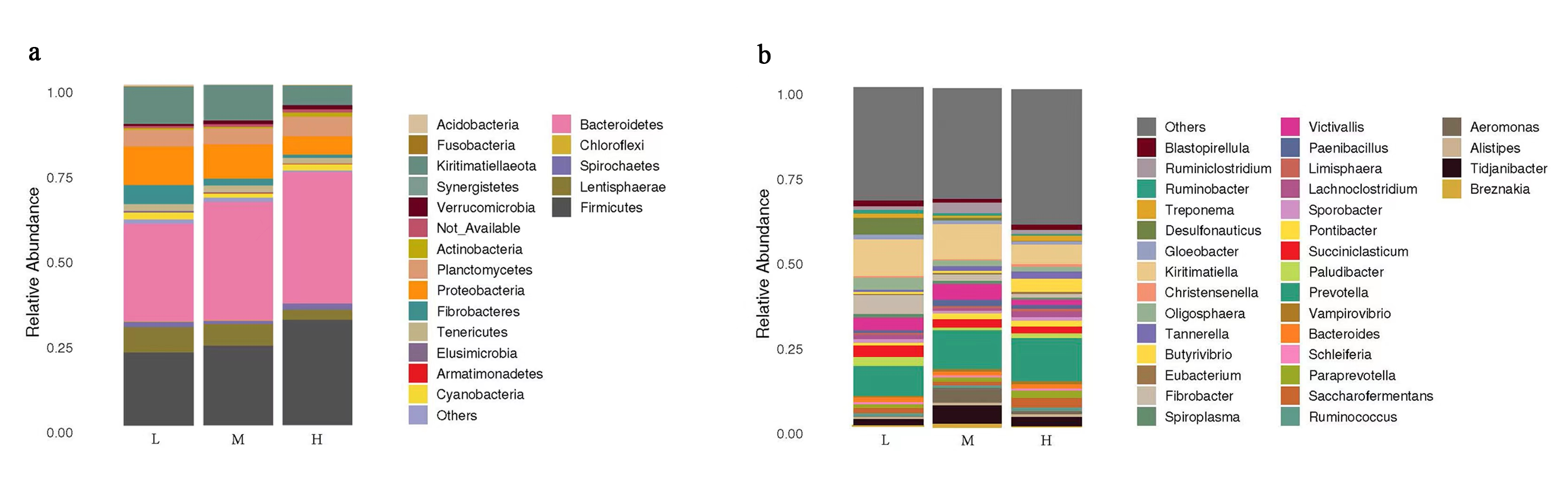


Supplementary Figure 1. (a)Phylum level. Bar chart of species abundance. (b) Genus level. The abscissa is the sample name and the ordinate is the relative abundance of species annotated. Those not annotated at this classification level were classified as Unclassified, and those species with abundance lower than 0.5% in all samples were classified as Others.


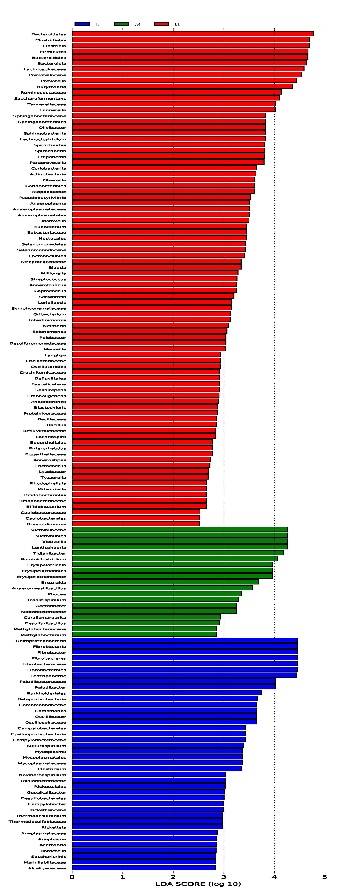


Supplementary Figure 2. LEfSe analysis of LDA plots. Different colors represent microbial taxa with significant effects in different groups. We mainly show the significant difference species with LDA score greater than the preset value, that is, the Biomaker with statistical difference, the default preset value is 2.0 (only the absolute value of LDA value greater than 2 will be shown in the Figure). The color of the bar graph represents the respective group, and the length represents the LDA score, which is the effect size of significantly different species between groups.
